# Genetic diversity, population structure and kinship relationships highlight the environmental influence on Uganda’s indigenous goat populations

**DOI:** 10.3389/fgene.2024.1385611

**Published:** 2024-05-30

**Authors:** Ziwena Nantongo, Josephine Birungi, Stephen Obol Opiyo, Gabriel Shirima, Swidiq Mugerwa, Collins Mutai, Martina Kyalo, Linus Munishi, Morris Agaba, Raphael Mrode

**Affiliations:** ^1^ Biosciences Eastern and Central Africa, International Livestock Research Institute, Consortium of International Agricultural Research Centers (CGIAR), Nairobi, Kenya; ^2^ School of Life Sciences, Nelson Mandela African Institution of Science and Technology, Arusha, Tanzania; ^3^ National Livestock Resources Research Institute, National Agricultural Research Organization, Kampala, Uganda; ^4^ Molecular and Cellular Imaging Center, The Ohio State University, Columbus, OH, United States; ^5^ Patira Data Science, Kampala, Uganda; ^6^ Scotland Rural College, Edinburgh, United Kingdom

**Keywords:** genetic diversity, population structure, indigenous goats, kinship relatedness, Uganda, Mubende goats, Kigezi goats, Small East African goats

## Abstract

Knowledge about genetic diversity and population structure among goat populations is essential for understanding environmental adaptation and fostering efficient utilization, development, and conservation of goat breeds. Uganda’s indigenous goats exist in three phenotypic groups: Mubende, Kigezi, and Small East African. However, a limited understanding of their genetic attributes and population structure hinders the development and sustainable utilization of the goats. Using the Goat Illumina 60k chip International Goat Genome Consortium V2, the whole-genome data for 1,021 indigenous goats sourced from 10 agroecological zones in Uganda were analyzed for genetic diversity and population structure. A total of 49,337 (82.6%) single-nucleotide polymorphism markers were aligned to the ARS-1 goat genome and used to assess the genetic diversity, population structure, and kinship relationships of Uganda’s indigenous goats. Moderate genetic diversity was observed. The observed and expected heterozygosities were 0.378 and 0.383, the average genetic distance was 0.390, and the average minor allele frequency was 0.30. The average inbreeding coefficient (Fis) was 0.014, and the average fixation index (Fst) was 0.016. Principal component analysis, admixture analysis, and discriminant analysis of principal components grouped the 1,021 goat genotypes into three genetically distinct populations that did not conform to the known phenotypic populations but varied across environmental conditions. Population 1, comprising Mubende (90%) and Kigezi (8.1%) goats, is located in southwest and central Uganda, a warm and humid environment. Population 2, which is 59% Mubende and 49% Small East African goats, is located along the Nile Delta in northwestern Uganda and around the Albertine region, a hot and humid savannah grassland. Population 3, comprising 78.4% Small East African and 21.1% Mubende goats, is found in northeastern to eastern Uganda, a hot and dry Commiphora woodlands. Genetic diversity and population structure information from this study will be a basis for future development, conservation, and sustainable utilization of Uganda’s goat genetic resources.

## 1 Introduction

Goats belong to the genus *Capra,* which is composed of nine species and includes *Capra hircus*, the domesticated goat. Domestic goats today are among the most essential livestock in developing countries, especially for the socio-economic, nutritional, and cultural roles in resource-poor farming households (Kaumbata et al., 2020). They are geographically widespread in most parts of the world, exhibiting a wide genetic diversity and agility in adapting to changing environments and demands. However, threats of biodiversity loss, climate change, and increasing human food demand (FAO. Africa Sustainable livestock, 2012) are a hindrance to the sustainable development of domestic goats. To ensure food security, especially animal protein foods, in the era of climate change, animal genetic resources within harsh environments will guarantee farmers and researchers the necessary flexibility in climate change adaptation (FAO and Commission on Genetic Resources for Food and Agriculture, 2007). Indigenous goats in Africa have been naturalized under harsh and diverse climatic conditions, and only those that were genetically adapted to the stressors survived and multiplied. Understanding the diversity of unique genetic characteristics of indigenous African goats and the population structure across various environments will significantly contribute towards the optimal utilization of these goat genetic resources for sustainable development. With a wider understanding of the population structure of goat genetic resources, researchers will have increased representation of diverse populations in large genome-wide association studies for leverage in discovering markers of economically important traits for food security and sustainability.

During domestication, ancestors of present-day domestic goat populations followed distinct dispersal routes along the east-west axis of Afro-Eurasia (Zheng et al., 2020). Goats encountered diverse environments along the domestication routes with unique climatic, anthropological, and biophysical limits to which they adjusted biologically or behaviorally for survival (Tarekegn et al., 2021). Behavioral adjustments resulted in short-term protection against environmental stressors, while biological adjustments gave long-term protection for survival through physiological and/or genetic adaptation. Because herders deliberately crossbred genetically adapted local populations with immigrant populations containing globally advantageous alleles, modern-day goat populations remain differentiated from each other (Zheng et al., 2020). Populations that are locally adapted to particular environments exhibit distinct observable characteristics that enable their survival under prevailing conditions and also assist in distinguishing among them as separate breeds. Currently, there are 1.2 billion goats in the world existing in over 1,000 breeds, and a significant majority (over 94%) are located in Asia (556 million heads) and Africa (388 million heads) (Nguyen et al., 2023), where they survive in very diverse environments. Most breeds from industrialized countries are well-defined phenotypically and are genetically distinct. In contrast, Asian and African breeds are most often local populations that differ only gradually according to geographical separation (FAO, 2011) but lack more information about their genetic uniqueness. Development of these local breeds is often ignored in favor of introducing exotic germplasm, about which more information is generally available (FAO, 2011).

Uganda is a landlocked country located in East Africa. It has over 16 million goats (UBOS, 2020), which are dominated by indigenous breeds phenotypically described as Mubende, Kigezi, and Small East African (FAO, 1991). The three breeds exist in proportions of 35.6%, 11.2%, and 53.2%, respectively (Ssewannyana, 2004). Mubende goats are known to have originated from the Mubende district (FAO, 1991; Mbuza, 1995) in the current western savannah grasslands zone, hence the breed name. They are large goats relative to Kigezi and Small East African goats, with an average live body weight of 31 kg for female goats and 35 kg for male goats. They are mainly black or black and white with a short, fine, shiny hair coat. Mubende goats are produced for meat, but their shiny hair coat is highly preferred in the leather tanning industry. Kigezi goats originate from the former Kigezi district in southwestern Uganda, currently called the Kabale district, in the highland ranges agroecological zone. They are distinguished from Mubende and Small East African goats by long, curly hair, especially on the hindquarters. Kigezi goats are moderate in size, with an average live body weight of 30 kg, and exist mainly in black or gray coat colors (FAO, 1991; Mbuza, 1995). Small East African goats include all the small goats that are not described as Mubende or Kigezi (Mbuza, 1995). They have an average live body weight of 25 kg and exist in multiple colors (FAO, 1991; Mbuza, 1995). Small East African goats are known to be hardy and able to survive under harsh climatic conditions with very low forage quality and quantity coupled with water scarcity (Mbuza, 1995). They are found in all areas of Uganda but are known to originate from northeastern Karamajong, Sebei, and Teso ecotypes and in northwestern areas around the Nile Delta (Onzima et al., 2017).

Indigenous goats in Uganda are known for low productivity, owing to slow growth rates and low mature weights despite being environmentally adapted for survival under prevailing conditions (Ssewannyana et al., 2004; Onzima et al., 2017). Due to the increasing demand for goat meat, efforts to improve the productivity of the indigenous goats across the country have focused on crossbreeding with exotic breeds like the Boer, Savannah, and red Kalahari (Ssewannyana, 2004). Over time, the inherent genetic characteristics of indigenous goats surviving in the different Ugandan environmental conditions may likely be lost before they are understood and conserved.

Investigations of genetic diversity, population structure, and demographic dynamics are investigated to understand inherent genetic differences among populations (Tarekegn et al., 2021). Single nucleotide polymorphism (SNP) genotyping technologies are the current popular tools used for animal genetic studies to understand diversity and population structure. They are also used in genome-wide association studies. The use of SNP genotyping technologies in goat genetics research became easier with the release of the Caprine SNP50 chip (Tosser-Klopp et al., 2014) developed by the International Goat Genome Consortium (IGGC) and its recent upgrade in collaboration with VarGoats Consortium to the Axiom Caprine Genotyping v2 Array to add more markers. Furthermore, the availability of the near complete goat genome (Li et al., 2021) further changed the status of genetics research in goats by providing a reference genome for clarity and consistency in SNP discovery and is a valuable resource for goat genetic research and applications. In this study, Uganda’s indigenous goats have been genotyped to understand the genetic diversity and population structure across all agroecological zones and to clarify the relationship between the phenotypic breed groups (Mubende, Kigezi, and Small East African) and their genetic identities.

The geographic patterns of genetic variation within modern populations, produced by complex migration histories, can be difficult to infer and visually summarize (Frichot et al., 2014). Thus, inference of individual ancestry coefficients is commonly performed using computer-intensive likelihood algorithms. The spatial non-negative matrix factorization (sNMF) algorithm helps to infer ancestral gene pools from genetic data. Entropy measures the level of disorderliness within the genetic data at particular points based on genetic compositions, and the level with minimum entropy is desired (Aning and Przybyla-Kasparek, 2022). By measuring the difference between the predicted and actual population labels, the cross-entropy criterion assists in optimizing the subgrouping process. Cross-entropy is helpful in choosing the number of ancestral populations that best explains the genotypic data. Given the absence of genetic sub-populations among Uganda’s indigenous goats plus the weak differentiation of the phenotypically labeled sub-populations (Onzima et al., 2018), unsupervised statistical methods were used to search the optimal number of genetic sub-populations and how to best group individuals based on their genetic identities.

Onzima et al. (2018) used the GoatSNP50Bead chip (Tosser-Klopp et al., 2014) to characterize the genetic diversity within and between five ethnically labeled indigenous goat populations in Uganda (Mubende, Kigezi, Small East African, Sebei, and Karamojong) and explored the extent of admixture of the Boer goat genetics among them. The authors found weak population sub-structuring among the indigenous goat populations, which was attributed to the recent establishment of these populations, possibly from the same founder population or closely related populations. Onzima et al. (2018) further explained that the small sample size used in their study was also likely unrepresentative of the populations and recommended that a large sample size and in-depth analysis of the goats’ history are required for proper understanding of the genetic diversity among Uganda’s indigenous goat populations. The GoatSNP50 Bead Chip (Tosser-Klopp et al., 2014) used in this study was developed from SNPs identified within and between six goat breeds (Alpine, Boer, Creole, Katjang, Saanen, and Savanna); the weak differentiation among Uganda’s indigenous goats could also likely be a result of ascertainment bias. With the upgrade of the GoatSNP50 Bead Chip to the Axiom Caprine Genotyping v2 Array, a 65K goat SNP bead chip that includes breeds from Africa and Uganda in particular (Denoyelle et al., 2021), it was important to clarify the diversity of Uganda’s indigenous goats using this high density and more inclusive SNP array. Furthermore, indigenous goats in Uganda survive in all agroecologies despite climatic and geographical differentiation, yet Onzima et al. (2018) only studied the goats in their known home areas. Understanding the spatial distribution of the different goat genetic populations in Uganda clarifies the influence of the environment on the goats, which is vital guidance to their sustainable utilization and conservation. This study examined the genetic diversity and population structure of Uganda’s indigenous goats across all agroecological zones using the Axiom Caprine Genotyping v2 Array to understand goats’ genetic diversity and distribution within and between the different environments.

## 2 Materials and methods

### 2.1 Sample collection

The study was conducted in 20 districts across the 10 agroecological zones of Uganda, as described in Nantongo et al. (2024). All goats selected for the characterization study were sampled using the Allflex tissue sampling technology (Allflex, 2021), and 1,036 ear tissue samples were collected. A unique barcode on each Allflex tissue sampling unit was recorded as the unique sample identification number for each sample. Details about the sampled animal, including animal identity, location, and phenotypic group identity, were then recorded under its unique identity (sample ID). All collected tissue samples were preserved at room temperature in preparation for laboratory analysis.

### 2.2 Genomic DNA extraction

Genomic DNA (gDNA) was extracted using a Maelstrom 9600 TANBEAD (Taiwan Advanced Nanotech Inc.) machine, following the tissue total DNA 6T2 protocol (Taiwan Advanced Nanotech, Inc., 2019) with some modifications. Approximately 100 mg of tissue sample was macerated and inserted into a 1.5-mL Eppendorf tube containing 200 µL of buffer RLT and 20 µL of proteinase K. The mixture was incubated using an Eppendorf thermomixer F1.5, 22,331 Hamburg model, set at 1,000 rpm and 56^o^ C overnight. A 200 μL aliquot of lysate was pipetted after spinning at 4,000 rpm in a centrifuge and added to the lysis plate of the 6T2 TANBEAD kit without the kit lysis buffer. The 6T2 kit protocol was modified to have 5 min activity time and 1000 rpm at the lysis plate, and the elution buffer was replaced with 60 µL of double-distilled water. Other DNA extraction steps followed the 6T2 protocol guidelines. The extracted genomic DNA was transferred to sample ID labeled 1.5-mL Eppendorf tubes. The quantity of gDNA extracted was estimated using NanoDrop spectrophotometry, and the quality assessment was done using gel electrophoresis.

### 2.3 Whole-genome SNP genotyping

A total of 1,036 genomic DNA samples were prepared and sent to LABOGENA in France, where whole-genome SNP genotyping was done based on the Goat Illumina 60k chip IGGC V2 containing 59,727 SNPs.

### 2.4 Data filtering and quality control

Data quality control was performed on 59,727 unfiltered SNPs that were received, and 53,327 had unknown chromosome positions. Chromosome positions for the entire dataset were updated using R software (version 4.2.3) based on the Goat Illumina 60k IGGC V2 (https://www.goatgenome.org/data/Goat_IGGC_65K_v2_15069617X365016_A2.csv) chip dataset whose SNP positions were aligned to the nearly complete ARS-1 goat genome (Li et al., 2021). Data were filtered in TASSEL (v5.2.87), where markers with a minor allele frequency (MAF) of less than 0.05, those with known chromosomes but no allocated chromosome positions, and markers whose chromosomes remained unknown after the update were excluded. Duplicated markers and genotypes with more than 20% missing data were also excluded.

### 2.5 Data analysis

#### 2.5.1 Kinship analysis

The kinship coefficient (KC) defines the likelihood of identity by descent of two homologous alleles drawn from different individuals (Speed and Balding, 2015). Kinship is half the additive genetic relationship among individuals. The extent of genetic relatedness among individuals was calculated using kinship coefficients based on the normalized identity-by-state method, which gives results similar to the traditional identity-by-descent (IBD) method (Nemesure et al., 1999). The expectation is that individual goats that are more related will share more alleles than non-related individuals. A kinship matrix based on a normalized identity-by-state (normalized-IBS) algorithm was generated across all genotypes using TASSEL (version 5.2.88). The generated kinship coefficients were translated into a kinship heat map in R (version 4.2.3) using the *gplots* package for easy visualization of the relationships.

### 2.6 Genetic diversity analysis

Estimating expected heterozygosity (He), observed heterozygosity (Ho), and MAF involves calculating the average frequency of alleles at each locus and comparing it with the actual frequency of alleles in the population. The resulting values provide valuable insights into the level of genetic diversity within the population. Evaluation of genetic diversity for all markers was done using the *hierfstat* package (Gruber et al., 2018) in R (version 4.2.3) as observed heterozygosity (Ho), expected heterozygosity (He), total heterozygosity (Ht), inbreeding coefficient (Fis), and fixation index (Fst). MAF and polymorphic information content (PIC) were estimated using the popgen function in the *snpReady* package (Granato and Fritsche-Neto, 2022).

### 2.7 Population structure analysis

Population structure refers to the patterns in neutral genetic variation that define the existence of differing levels of genetic relatedness among some subgroups within a sample. To ensure neutral genetic variation, Hardy–Weinberg analysis was done on all markers in R (version 4.2.3) using the Hardy–Weinberg test in the *pegas* package. Statistically significant test results are suggestive of deviation from the Hardy–Weinberg equilibrium (HWE) assumption; thus, SNPs with a probability of a chi-squared test result (Pr (<chi^2 >) of less than 0.05 were removed from the dataset. Markers in the HWE were used to assess the genetic population structure through admixture analysis, principal component analysis, and discriminant analysis of principal components.

### 2.8 Admixture analysis

In an attempt to estimate the effective number of populations in the dataset, an analysis of population structure was done based on admixture analysis (Liu et al., 2020) using the *landscape and ecological association (LEA)* package in R (4.2.3) with 10 estimated ancestral populations (k), 10 iterations/runs for each k, and 100,000 repetitions. The effective number of populations was estimated using the cross-entropy criterion based on the elbow method. The extent of admixture of the populations was illustrated with a structure graph, and inferred populations were colored differently for easy visualization. Genotypes presenting 50% or more ancestral proportions for a population were clustered together as representatives of that population. For genotypes within each formed cluster, the phenotypic breed label and location of sample collection were attached to understand the distribution of the genotypes across breed labels and localities. The distribution of the inferred populations across localities was also graphically illustrated. To further explore the extent of genetic relatedness within and between the inferred genetic populations, kinship coefficients for all genotypes were calculated based on normalized identity by state algorithm and compared within and between clusters.

### 2.9 Principal component analysis

Principal component analysis (PCA) uses the dimensionality reduction method to present the maximum amount of variation, which is stored in new uncorrelated variables called principal components (Liu et al., 2020). To further understand the population structure of the phenotypically classified indigenous goat breeds in this study, PCA was done using the *LEA* package (Patterson et al., 2006). The Tracy–Widom test was done to understand the significant eigenvalues and the percentage contribution of each principal component. The distribution of the individual goats in their phenotypically defined breeds was laid out in a 2D PCA variance plot using the *ggplot2* package from R (v 4.3.2) to understand their genetic diversity distribution along the major principal components. Furthermore, a 3D variance plot was generated from the first three major principal components using the *rgl* package from R (v4.3.2) to clarify the real positioning of the individuals.

### 2.10 Discriminant analysis of principal components (DAPC)

In order to visualize the complex population structure, test assignment of individuals to clusters, and identify genomic regions driving population differences, discriminant analysis of principal components (DAPC) (Jombart et al., 2010) was done using HWE markers using the *adegenet* package in R (version 4.2.3). Cross validation analysis was done to find the appropriate number of principal components that give the highest successful estimation at the lowest random error. A DAPC cross-validation plot was generated to visualize the appropriate number of principal components. Discriminant analysis of principal components was done, and a scatter plot of the two major principal components of the DAPC was generated to visualize the distribution of the breed groups. The markers mainly contributing to the separation of the breed clusters were visualized through plots of loadings for each of the major principal components.

## 3 Results

**FIGURE 1:**
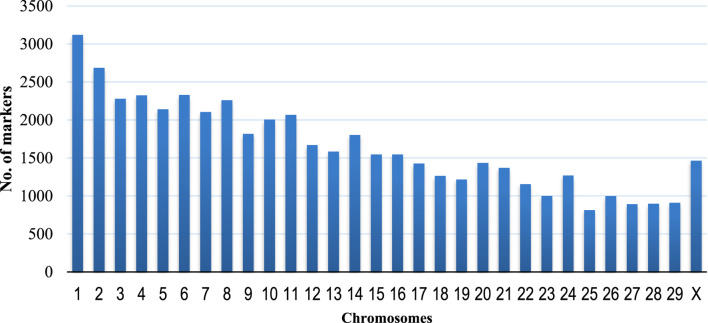
Distribution of the 49,366 markers along the chromosomes of *Capra hircus*.

A total of 59,727 unfiltered SNPs were generated after the Goat Illumina 60 k chip IGGC V2 genotyping of 1,032 goats from Uganda. A total of 15 genotypes and 10,361 SNPs (Table 1) (17.3%) were excluded (6,243 SNPs had MAF below 0.05, and 4,118 SNPs had unknown chromosomes or chromosome positions). The final dataset contained 1,021 genotypes and 49,366 markers distributed across chromosomes of *C. hircus* after data quality control and filtering. The highest number of SNPs were found on chromosome 1 (6.32%), followed by chromosome 2 (5.44%) and chromosome 6 (4.72%), while the lowest number of SNPs were on chromosome 25 (1.65%) (Figure 1). A total of 1,021 goats were retained after filtering for genotypes with more than 20% missing data.

**TABLE 1:** Number of SNPs or genotypes excluded after quality control.

Quality control parameter	SNPs	Genotypes
Number before quality control	59,727	1,032
SNPs with unknown chromosomes/chromosome positions	4,118	-
SNPs with MAF below 0.05	6,243	-
Genotypes with more than 20% missing data	-	11
Number after quality control	49,366	1,021

### 3.1 Genetic diversity across the indigenous goat genotypes and SNP markers studied

**FIGURE 2:**
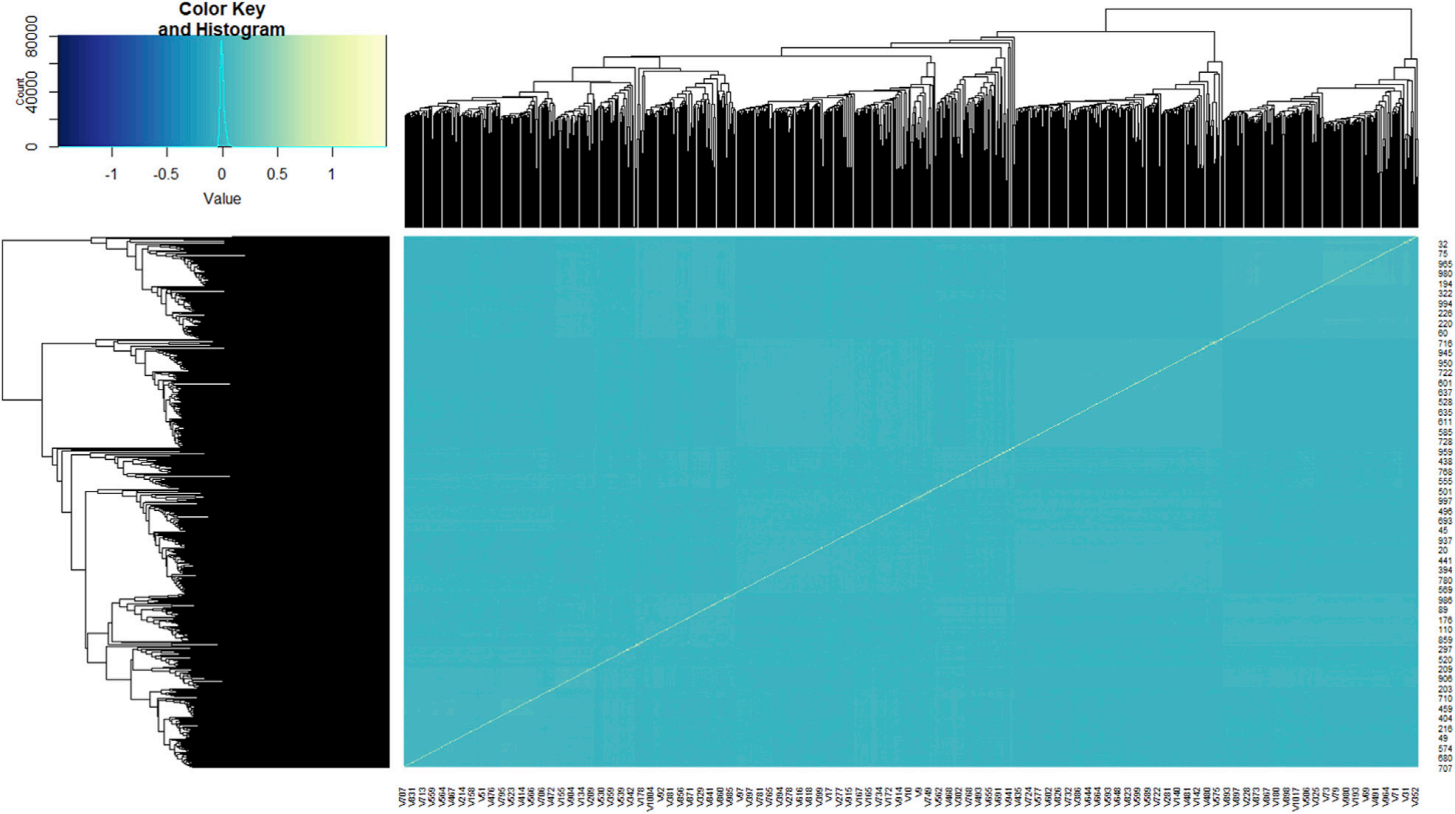
Kinship heat map of the 1,021 goat genotypes studied. Each pixel of the heat map shows the strength of the correlation between the individuals, with yellow indicating a strong correlation and blue indicating no correlation.

In general, the goats clustered into three major groups (Figure 2). The kinship coefficient between pairs of goat genotypes ranged from −0.057 to 1.486, inferring the extent of allele sharing among them. The average genetic distance between genotypes was 0.390, ranging between 0 and 0.429. Across the 49,366 markers, overall estimates of observed heterozygosity (H_o_), expected heterozygosity (H_e_), and total heterozygosity (H_t_) were 0.378, 0.383, and 0.390, respectively. The average inbreeding coefficient (Fis) was 0.014, indicating slight (1.4%) homozygosity, while the average fixation index (Fst) was 0.016, which showed that 98.4% of the variation was within populations. MAF ranged between 0.00 and 0.50 with an average MAF of 0.30, while PIC ranged between 0.00 and 0.38 with an average PIC of 0.31.

### 3.2 Principal component analysis

**FIGURE 3:**
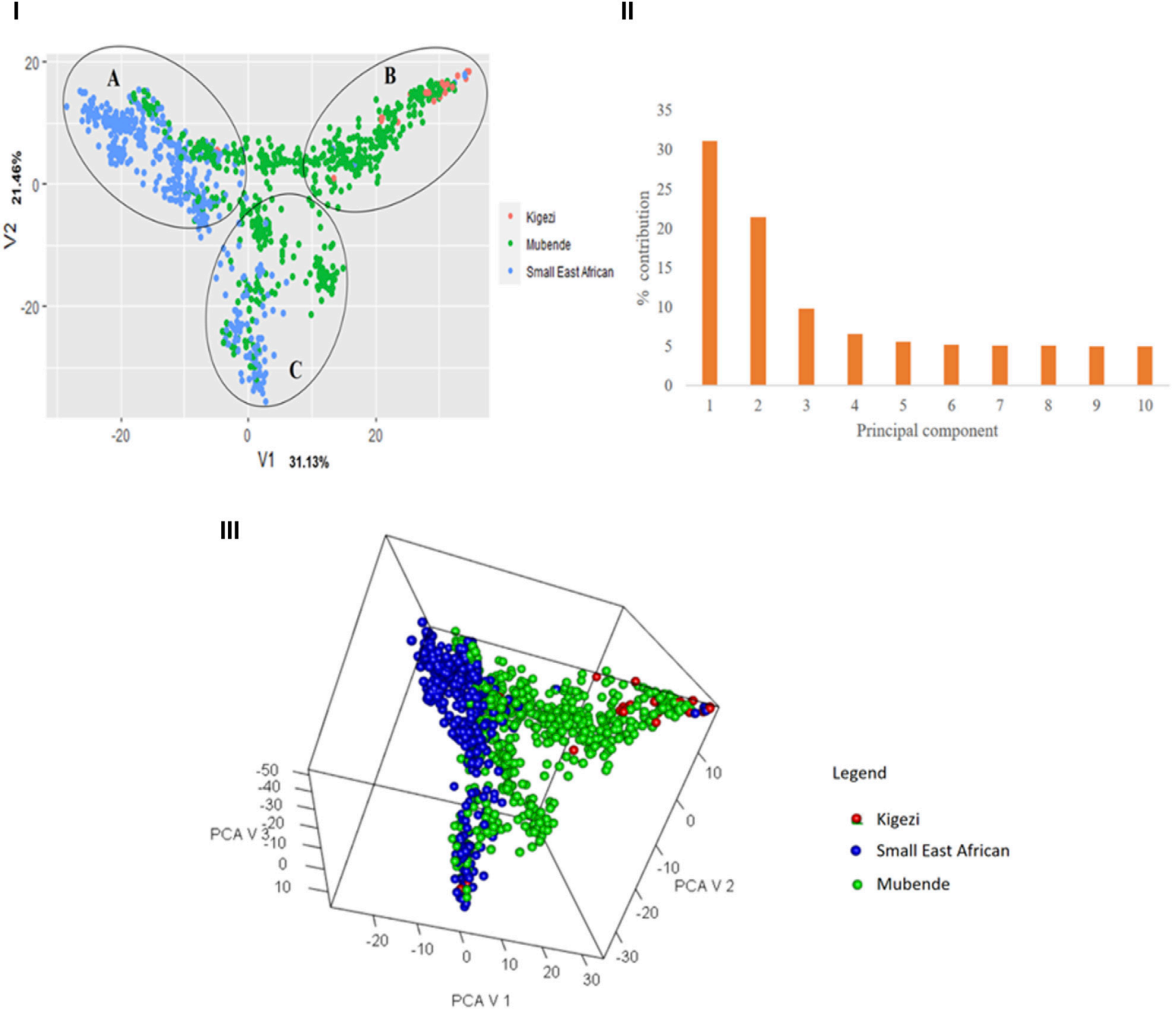
Variance plot showing the distribution of individuals across the major principal components (I), a scree plot with the percentage contribution of different components to the variation observed (II), and a 3D plot to show the relative positions of the individuals based on genetics. Note: Individuals have been labeled with their phenotypic population groups for a comparative view.

The first principal component, V1, explained 33.4% of the variation, followed by V2, which explained 21.2% (Figure 3I). Cumulatively, the two major components explained 52.5% of the variation, and 80% of the variation was explained by the first six principal components (Figure 3II). The 3D variance plot of the first three principal components clustered the individuals into three groups and showed that genetic population groups do not follow the phenotypic breed groupings (Figure 3III). Cluster A comprises mainly Small East African goats but has a proportion of Mubende goats. Cluster B mainly consisted of Mubende goats and was mixed with most of the Kigezi goats obtained in the study. Cluster C is a mixture of Mubende and Small East African goats.

### 3.3 Population structure

**FIGURE 4:**
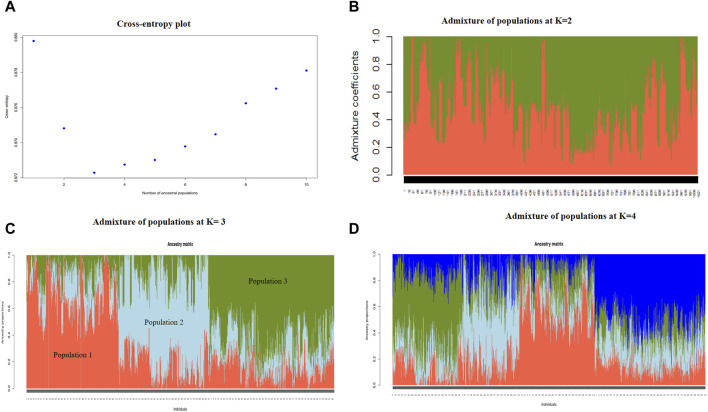
Cross-entropy plot showing the optimal number of ancestral populations at the elbow point **(A)** and the extent of admixture of populations at K = 2 populations **(B)**, K = 3 populations **(C),** and K = 4 populations **(D)**.

To understand the population structure of indigenous goats in Uganda, the entropy criterion was used to estimate the number of ancestral populations that best explain the genotypic data. Based on the elbow method at the cross entropy, three ancestral populations are depicted (Figure 4A), which is consistent with the results of the PCA analysis. The genetic structure at the K = 2, K = 3, and K = 4 populations have been graphically presented to show the extent of admixture at the inferred ancestral populations (Figures 4B–D). At K = 3, the three populations with individuals sharing more than 60% ancestral proportion are clearly observable. Genotypes in Population 1 have about 80% ancestral proportions for Population 1, 10% for Population 2, and 10% for Population 3. Population 2 genotypes have 70% ancestral proportions for Population 2, 20% for Population 1, and 10% for Population 3, while Population 3 genotypes are about 75% Population 3, 5% Population 2, and 20% Population 1.

Upon relating the genotypes in each cluster to the ascribed phenotypic goat breeds, the 309 genotypes in Cluster 1 included 279 Mubende (90.3%), 25 Kigezi (8.1%), and five Small East African (1.6%) goats; the 305 genotypes in Cluster 2 included 180 Mubende (59.0%) and 125 Small East African (41.0%) goats, and the 407 genotypes in Cluster 3 include 86 Mubende (21.1%), 2 Kigezi (0.5%), and 319 Small East African (78.4%) goats. In relation to the breed distribution in the PCA plot (Figure 3) above, Cluster 1 corresponds to Cluster B, Cluster 2 corresponds to Cluster C, and Cluster 3 corresponds to Cluster A.

**FIGURE 5:**
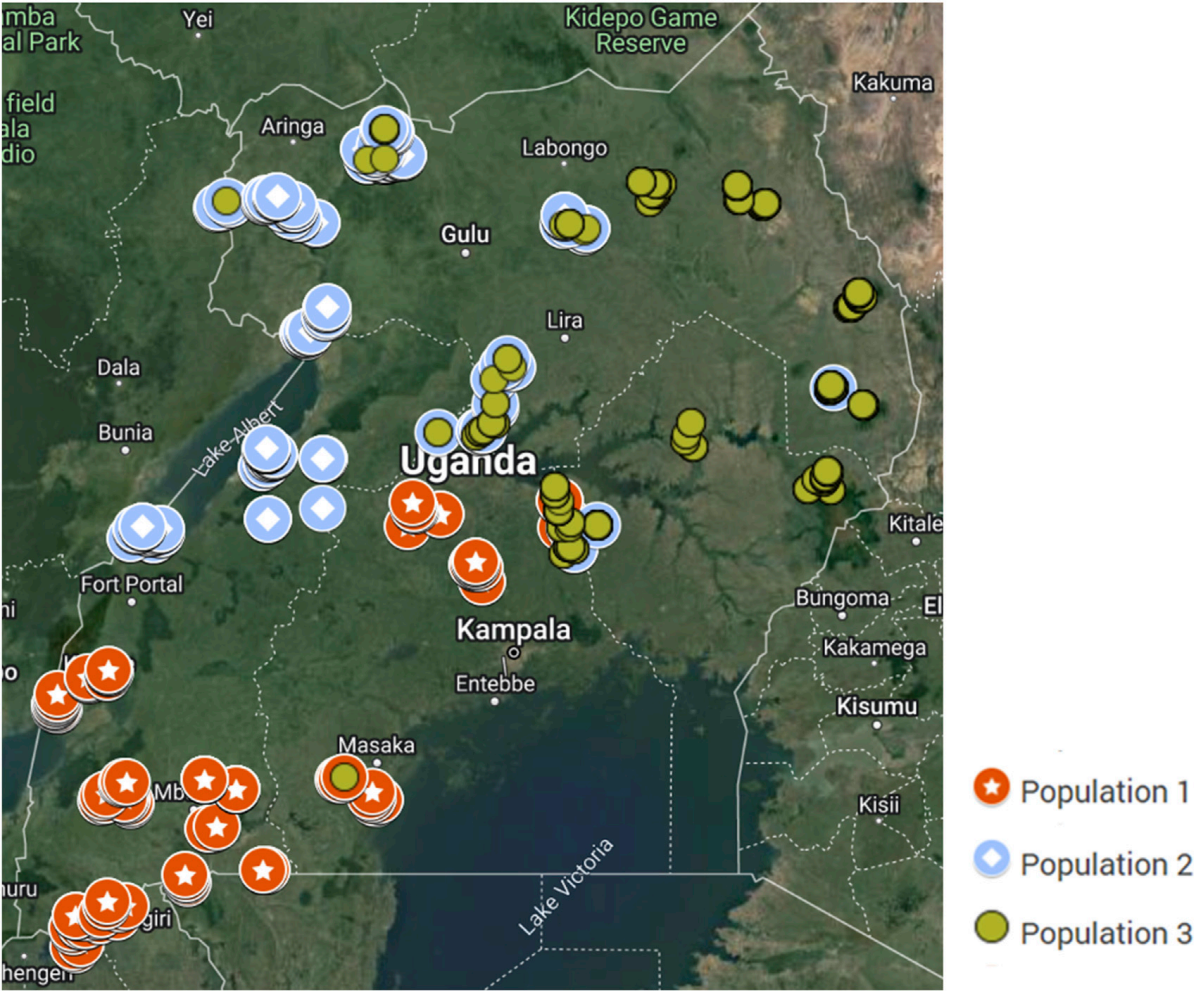
Map of Uganda showing the geographical distribution of the inferred indigenous goat populations.

A tendency of geographical distribution of the populations was observed (Figure 5). Population 1 genotypes are mainly distributed in localities within the warm and humid central to southwestern Uganda. Genotypes in Population 2 are mainly found in the hot and humid areas around the Nile, Lake Albert, and Lake Kyoga. Genotypes in Population 3 are distributed mainly in the hot and dry areas in the Karamoja region, towards northern and eastern Uganda.

### 3.4 Kinship relatedness of individuals within and between the three inferred populations

The average kinship coefficients for goats within and between Clusters 1, 2, and 3 are presented in Table 2. The kinship coefficient within clusters varies from 0.0150 ± 0.037 for Cluster 3 to 0.0228 ± 0.038 for Cluster 1 genotypes. Between-cluster KC varied from −0.0128 ± 0.037 for Clusters 1 and 3 to −0.0061 ± 0.038 for Clusters 1 and 2. Therefore, individuals within each cluster are more related to each other than to individuals in different clusters.

**TABLE 2:** Means, standard deviations, and ranges (in parentheses) of kinship coefficients within and between clusters.

	Cluster 1	Cluster 2	Cluster 3
Cluster 1	0.0228 ± 0.038 (−0.033–1.335)	−0.0061 ± 0.038 (−0.042–0.065)	−0.0128 ± 0.037 (−0.059–0.579)
Cluster 2		0.0156 ± 0.037 (−0.031–1.464)	−0.0071 ± 0.036 (−0.046–0.306)
Cluster 3			0.0150 ± 0.037 (−0.039–1.337)

To further understand the relatedness of the different phenotypically labeled goat breeds that are within the same cluster, the average kinship coefficients and ranges were calculated within and between the breeds within each of the clusters (Table 3). Again, the within-cluster averages between phenotypically labeled breeds were positive and ranged from 0.01 to 0.064, which represents some genetic additive relationship equivalent to progeny and great-grand-parent relationship. In addition, the average kinship coefficients were negative to almost zero between different clusters.

**TABLE 3:** Means, standard deviations, and ranges (in bracket) of kinship coefficients within and between phenotypic breed labels of goats across clusters.

		Cluster 1			Cluster 2		Cluster 3	
		Mubende	Kigezi	Small East African	Mubende	Small East African	Mubende	Small East African
Cluster 1	Mubende	0.021 ± 0.063 (−0.033 - 1.335)	0.028 ± 0.024 (−0.027 - 0.211)	0.025 ± 0.022 (−0.027 - 0.114)	−0.003 ± 0.011 (−0.039–0.065)	−0.010 ± 0.009 (−0.042–0.061)	−0.006 ± 0.012 (−0.052–0.579)	−0.014 ± 0.010 (−0.059–0.121)
	Kigezi		0.099 ± 0.188 (−0.017–1.158)	0.064 ± 0.075 (−0.007 - 0.509)	−0.007 ± 0.011 (−0.038–0.038)	−0.016 ± 0.008 (−0.039–0.035)	−0.012 ± 0.011 (−0.048–0.033)	−0.020 ± 0.010 (−0.056–0.056)
	Small East African			0.288 ± 0.381 (0.005–1.072)	−0.006 ± 0.012 (−0.039–0.035)	−0.014 ± 0.009 (−0.038–0.034)	−0.011 ± 0.012 (−0.045 - 0.043)	−0.019 ± 0.010 (−0.055–0.054)
Cluster 2	Mubende				0.017 ± 0.079 (−0.028–1.464)	0.010 ± 0.016 (−0.031 –0.306)	−0.007 ± 0.010 (−0.044–0.053)	−0.008 ± 0.011 (−0.042–0.066)
	Small East African					0.029 ± 0.091 (−0.020–1.262)	−0.008 ± 0.010 (−0.046–0.104)	−0.005 ± 0.012 (−0.046–0.306)
Cluster 3	Mubende						0.022 ± 0.116 (−0.025–1.337)	0.008 ± 0.011 (−0.036–0.292)
	Small East African							0.019 ± 0.059 (−0.039–1.284)

### 3.5 Discriminant analysis of principal components

**FIGURE 6:**
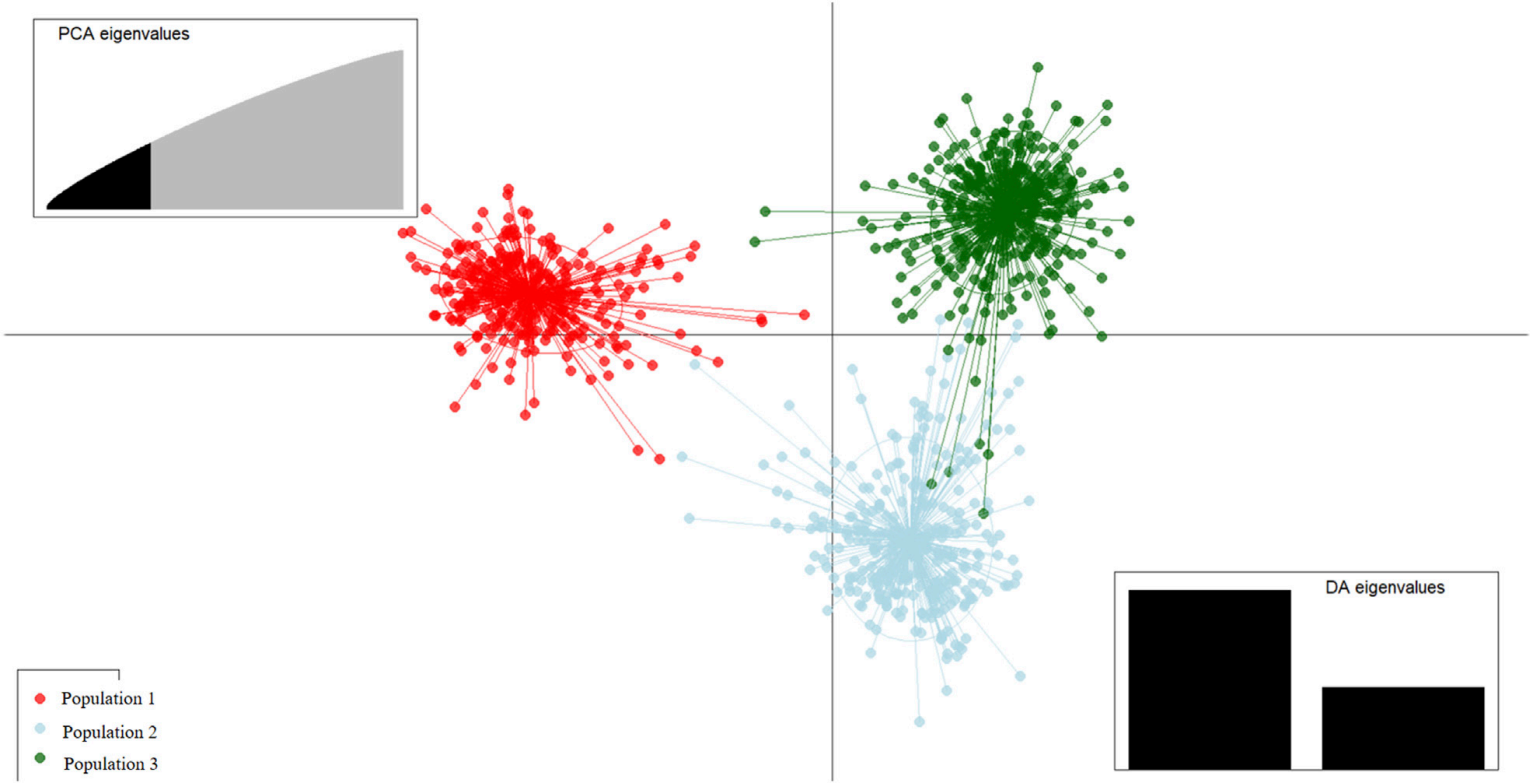
Scatterplot of the first two principal components from discriminant analysis of principal components (DAPC) discriminating indigenous goats of Uganda by the population structure Clusters 1, 2, and 3. Points represent individual observations; lines and colors represent group membership.

Discriminant analysis of principal components (DAPC) is a multivariate model-free approach to clustering based on prior population information. The genetic clusters formed from admixture analysis were subjected to DAPC to assess how well the individuals could be reassigned to their clusters/populations. The extent of divergence of each cluster from another was also observed. Based on the cross-validation procedure, 300 principal components were retained for accurate DAPC estimation. Generally, each cluster is distinctly separated from another, indicating that the three are genetically different populations. Along the first principal component, Cluster 1 individuals were clearly separated from Clusters 2 and 3 (Figure 6), while the second principal component separated Cluster 2 from both Cluster 1 and Cluster 3. This distinction was reflected in the percent correct posterior assignment of the goats to original populations/clusters. Overall, there was a 98.3% assignment-success rate. Population 1 received 99.68% successful reassignment, Population 2 received 96.72% successful reassignment, and Population 3 received 98.53% successful reassignment. The final group sizes for each population after DAPC were 311 goats in Population 1, 300 in Population 2, and 410 in Population 3.

Variation along the first principal component clearly distinguishes Population 1 from Population 2 and Population 3 goats (Figure 6), and the markers that are mainly involved are shown in (Table 4). The second principal component distinguishes Population 2 clearly from Population 1 and Population 3, and the markers involved are shown in Table 5.

**TABLE 4:** Markers mainly involved in the separation of Population 1 from Population 2 and Population 3 along the first principal component.

Marker	Chromosome no.	Chromosome position	Map information
snp59186-scaffold971-1067105	1	143214056	145,490,320
snp39461-scaffold501-1021148	10	19,327,578	81,628,205
snp5329-scaffold1,184-105727	14	77,782,362	14,743,386
snp50397-scaffold720-707496	17	4,0,871,919	29,929,553
snp10217-scaffold1,368-404221	23	42,911,082	4,614,248
snp1756-scaffold1049-548491	24	25,594,404	25,783,341

**TABLE 5:** Markers mainly involved in the separation of Population 2 from Population 1 and Population 3 along the second principal component.

Marker	Chromosome no.	Chromosome position	Map information
snp35792-scaffold430-2747761	2	40,629,104	94,947,889
snp54328-scaffold83-2242945	3	74,492,290	42,858,734
snp39013-scaffold494-5513280	4	74,850,129	42,880,995
snp57324-scaffold912-2828826	10	46,199,809	53,162,170
snp3913-scaffold1,122-1834494	9	23,679,741	23,877,069
snp36846-scaffold447-2328534	9	81,076,900	82,207,848
snp19625-scaffold1983-135219	14	3,686,372	90,967,963
snp8916-scaffold1,320-247796	21	28,452,211	30,215,839
snp688-scaffold102-2306174	24	21,199,606	21,388,595
snp689-scaffold102-2349633	24	21,243,065	21,431,890
snp13113-scaffold1,501-193208	X	106,377,986	44,169,996
snp53009-scaffold796-1480034	29	27,844,650	28,315,349

## 4 Discussion

Knowledge about genetic diversity and population structure among goat populations is essential for understanding environmental adaptation and fostering efficient utilization, development, and conservation of goat breeds. This study characterized 1,021 indigenous goats from 10 agroecological zones in Uganda to fill the information gap about the genetic diversity and population structure of Uganda’s indigenous goats across agroecological zones. The diverse husbandry and agroeco-climates across the agroecological zones appear to influence the genome architecture of Uganda’s indigenous goats. In general, all goats studied had low genetic relatedness and high genetic diversity. The observed and expected heterozygosity of more than 0.37 and the average genetic distance of 0.390 indicate that Uganda’s indigenous goats are genetically diverse. Similar values of heterozygosity were observed among indigenous goats in Ethiopia (Tarekegn et al., 2021), Cameroon (Tarekegn et al., 2019), China (Berihulay et al., 2019), and Mongolia (Mukhina et al., 2022). In addition, low and positive values of F_IS_ and F_ST_ obtained in this study indicate high heterozygosity and no deliberate inbreeding in the goat population, which limits the impact of deleterious alleles, inbreeding depression, and loss of variance (Berihulay et al., 2019). The average PIC of 0.31 indicates that markers used in this study were informative and are, therefore, useful in assessing the genetic variation within and among indigenous goat populations of Uganda.

The analysis of population structure based on SNPs provides helpful information in maintaining and monitoring the genetic diversity required for a robust breeding program. A population’s genetic structure is determined by the interaction of processes such as gene flow, mutation, selection, and mating strategy (Muriira et al., 2018). The population structure of Uganda’s indigenous goats has, for the first time, been analyzed based on whole genome SNP genotyping with the Goat_IGGC_65K_v2 SNP chip (Tolone et al., 2022). The genetic population structure was assessed through PCA, admixture analysis, and discriminant analysis of principal components (DAPC). Three genetically distinct goat populations were inferred with a geographical distribution. All goats in southwestern to central Uganda clustered into Population 1, which contains most of the phenotypically labeled Mubende goats and all the Kigezi goats. Individuals in Population 2 are found in northwestern Uganda along the Nile basin towards the Albertine region; they are a mix of phenotypic Mubende and Small East African goats. Population 3 is mainly found in the northeast, especially in the Karamoja region and parts of eastern Uganda, with mostly the phenotypic Small East African goats. Although Kigezi and Mubende may appear phenotypically different, genetically, they are one population. The findings contradict Onzima et al. (2018), probably because the ethnic and phenotypic groups were considered as breeds. In this study, phenotypic breed labels were not used to infer population structure; rather, the genetic variability of individuals was used. This study shows that the grouping of the indigenous goat population according to phenotypic appearances and ethnic relationships is not consistent with genetic groupings. Three genetically distinct populations were inferred, conforming more to their ability to adapt rather than phenotypic appearance. This is further confirmed by results of kinship analysis that showed less genetic relatedness for the goats of the same phenotypic breed label in different genetic populations and more genetic relatedness for those of different phenotypic labels in the same genetic population.

The observed distribution of the three genetic populations in the different parts of Uganda is probably a reflection of the populations’ adaptation to survival in the different environments. Goats of Population 1 are mainly distributed in the southwest towards central Uganda; Population 2 is found in the northwest and along the Nile Delta area, while Population 3 is mainly found in northeastern to eastern Uganda. Environmental differences in the three sections of the country appear to play a vital role in shaping the genetic structure of these indigenous goats. Agroecological zones in southwestern to central Uganda have savannah vegetation characterized by acacia shrubs (Onzima et al., 2017) in a warm-humid climate (MAAIF, 2018), which may be more favorable for the survival of Population 1 goats. Population 1 is dominated by the Mubende phenotypic group of goats, which are described as relatively larger in size than other indigenous goats in Uganda (FAO, 1991; Nantongo et al., 2024) and predominantly black in color (FAO, 1991). Population 2 goats in northeastern Uganda thrive in a hot and humid environment with limited land available for grazing (Onzima et al., 2017), hence limited feed availability. The goats from agroecological zones in northeastern Uganda are the smallest in body size compared to indigenous goats in other agroecological zones (Nantongo et al., 2024). This may be associated with their ability to adapt to the prevailing environmental conditions. Indeed, body conformation in terms of size and shape is considered one of the morphological characteristics for higher adaptive and resilient capacity under different climatic zones (Ramachandran and Sejian, 2022). Similarly, the hot and dry environmental conditions in eastern to northeastern Uganda may influence the genetic composition of goats in Population 3. A clear understanding of the population structure of indigenous goats in Uganda is vital to guide efforts in their breeding, conservation, and allocation to appropriate environments for efficient productivity and sustainability.

## 5 Conclusions and recommendations

The genetic diversity and population structure of indigenous goats in Uganda were characterized, and the indigenous goat populations were grouped based on their genetically ascribed characteristics for the first time. There is moderate genetic diversity among the indigenous goats, and this needs to be conserved and appropriately utilized. Uganda has three genetically distinct goat populations with a geographical structure. The identified goat populations do not conform to the existing phenotypic/ethnic breed identities; the study has shown that Kigezi and Mubende ascribe to the same genetic group (Population 1), while the Small East African from northwestern and northeastern Uganda form two distinct genetic groups (Populations 2 and 3, respectively). Genetic associations with environmental adaptability rather than phenotypic appearance have been shown to be an important factor in Uganda’s indigenous goat population stratification. There is, therefore, a need to reconsider the breed identification for Uganda’s indigenous goats such that genetically distinct individuals are phenotypically identifiable and labeled as distinct populations. Such an exercise of identifying breeds on the basis of genotyping would be expensive. Therefore, incorporating genotypic information into the design of breed improvement programs is essential, and genetic differences are accounted for. Information from this study will be useful in designing breed improvement strategies for indigenous goats in Uganda and guide the planning of conservation for goat genetic resources in Uganda, which will lead to sustainable utilization. Finally, information from this study can be used to add value to the understanding of the entry and dispersal of goats to the African continent from the Southwest Asian domestication center (Gifford-Gonzalez and Hanotte, 2011), where the existing map has no route showing entry and dispersal of goats in Uganda.

## Data Availability

The original contributions presented in the study are publicly available. This data can be found here: 10.5061/dryad.w0vt4b910.
